# Politeness cannot make up for robots’ errors

**DOI:** 10.3389/frobt.2026.1760163

**Published:** 2026-06-17

**Authors:** Shikhar Kumar, Eliran Itzhak, Noam Tractinsky, Galit Nimrod, Vardit Sarne-Fleischmann, Yael Edan

**Affiliations:** 1 Department of Industrial Engineering and Management, Ben-Gurion University of the Negev, Be’er Sheva, Israel; 2 Department of Software and Information Systems Engineering, Ben-Gurion University of the Negev, Be’er Sheva, Israel; 3 Department of Communication Studies, Ben-Gurion University of the Negev, Be’er Sheva, Israel; 4 Department of Social & Health Sciences, Bar Ilan University, Ramat Gan, Israel

**Keywords:** assistive robot, human-robot interaction (HRI), politeness, robot errors, types of robots

## Abstract

We investigated the impact of robot politeness and error-prone behavior on user perceptions through two user studies involving non-humanoid robots. Politeness was operationalized at two levels based on Lakoff’s, (1973) politeness rules—one condition implemented all three of Lakoff’s rules, demonstrating the highest level of politeness, while the other omitted them, resulting in a behavior that was strict but not impolite. The correctness was manipulated by comparing an error-free robot behavior to a behavior that included intentional errors. The studies were conducted using two different tasks with two robot types—a mobile robot and a manipulator robot—and involved 59 young adult participants (ages 24–28) with engineering backgrounds. Participants consistently rated the correct and polite robots most favorably. However, politeness did not offset the negative effects of erroneous behavior. In both studies, the robot that was correct but strict was rated more positively than the one that was polite yet made mistakes. These findings suggest that, at least in utilitarian task settings involving technically proficient users, politeness alone cannot compensate for performance failures. Moreover, a polite robot that makes errors may even frustrate users more than a straightforward but accurate one. The findings also emphasize the importance of evaluating HRI performance across different robot types and tasks, as these factors significantly shape user perceptions.

## 1 Introduction

Robots possessing high-quality social skills can improve human-robot collaboration and interaction (Loi et al., 2018; García-Soler et al., 2018; Jo and Hong, 2025). One such skill is politeness, an important aspect of human-human interaction that helps people manage smooth interactions, maintain positive relationships, reduce conflicts among interacting individuals (Bar-Or et al., 2022), and enhance the effectiveness of the interaction. The media equation theory (Reeves and Nass, 1996) proposes that people respond to computers the same way they respond to humans. Accordingly, incorporating politeness into human-computer interaction (HCI) or human-robot interaction (HRI) may yield similar results to those in human-human interaction. Nass (2004) reported that people tend to behave politely toward the computer once it shows polite behavior. However, early research on HCI (Whitworth, 2005; Whitworth and Liu, 2009; Meyer et al., 2016; Hayes and Miller, 2010) has addressed the question of polite interaction between humans and computers only conceptually, without the thorough empirical examination required. Incorporating computer politeness improves trust in the system (Miller, 2005; Parasuraman and Miller, 2004). It could provide the user with a better understanding of the automation system (Lee and See, 2004) and help eliminate the misunderstanding between the automation system and its users (Lee and See, 2004). More recent work [e.g., (Inbar and Meyer, 2019; Salem et al., 2014; Salem et al., 2013; Ritschel et al., 2019; Zhu and Kaber, 2012; Kumar et al., 2021; Kumar et al., 2022a; Kumar et al., 2022b)] has contributed mixed empirical findings on politeness in HRI, which will be reviewed below in Section 2.

Politeness in the field of HRI has been dominated by research with humanoid robots (Inbar and Meyer, 2019; Salem et al., 2014; Salem et al., 2013; Ritschel et al., 2019; Zhu and Kaber, 2012). However, non-humanoid robots are widely used across both industrial Lim (2025) and non-industrial domains (Bona et al., 2014), fulfilling roles in a wide range of professional and personal services (Esterwood et al., 2024; Cha et al., 2018; Coeckelbergh, 2011). In non-industrial contexts, professional service robots operate in urban environments to support public services, transportation, and logistics (e.g., autonomous delivery vehicles or warehouse robots) (Nagenborg, 2020; Olatunji et al., 2019). They also assist in healthcare settings, such as medication delivery or patient monitoring (Robinson et al., 2014). In personal service applications, non-humanoid robots can function as assistive technologies in the home (e.g., robotic vacuum cleaners, elder care robots) (Balaguer et al., 2005), and sometimes as hybrids combining assistive and social features (Esterwood et al., 2024; Rudall, 2004).

Non-humanoid robotic systems typically include two types: manipulator robots and mobile robots. Manipulator robots are often stationary and designed to perform tasks using articulated arms—common examples include industrial robotic arms used for assembly, packaging, or sorting of items. Mobile robots, in contrast, are capable of autonomous navigation and are used in tasks such as indoor delivery (e.g., robots in hospitals), surveillance (e.g., robotic patrol units), or household cleaning (e.g., Roomba).

Since many robotic applications involve non-humanoid robots, it is essential to extend research on politeness in human-robot interaction (HRI) to such systems, as polite behavior may foster more positive emotional responses from users. Previous studies (Kumar et al., 2021; Kumar et al., 2022a; Kumar et al., 2022b) examined politeness in interactions with non-humanoid robots. In particular (Kumar et al., 2021; Kumar et al., 2022b) investigated three levels of politeness during interactions with both a non-humanoid manipulator and a mobile robot. Across 203 participants from younger and older age groups, the findings showed that politeness enhanced user perceptions; however, participants could reliably distinguish only two of the three polite levels. A subsequent study focusing on older adults (Kumar et al., 2022a), examined the influence of culture and gender on perceptions of robot politeness using a manipulator robot (Kumar et al., 2022a). The results revealed cultural differences and underscored the importance of examining these factors in broader age groups.

In this paper, we present a user study that examines the effects of politeness and erroneous robot behavior on user perceptions during interactions with two types of non-humanoid robots: a mobile robot and a manipulator. To better reflect real-world conditions in which robots frequently encounter errors, the study explicitly incorporates an error construct into the experimental design (Honig and Oron-Gilad, 2018). The study focuses on young adult participants.

Errors can heavily affect user experience (UX) in interaction with robots (Honig and Oron-Gilad, 2018; Salem et al., 2015; Rossi et al., 2023; Flook et al., 2019) as in conventional computer interaction (Do et al., 2023). Yet, there is limited research on how an agent should phrase its response once something goes wrong (Spitale et al., 2024). Work in human-AI interaction shows that thoughtful recovery strategies, such as explanations or apologies, can restore (Honig and Oron-Gilad, 2018) users’ perceptions of an agent’s intelligence, likability, and competence after a failure. By contrast, (Lee et al., 2019) found that politeness was considered useless in critical working situations where immediate failure recovery is needed.

In this paper, we examine politeness and error together rather than in isolation. Specifically, we investigate whether and when adherence to politeness maxims facilitates a robot’s ability to maintain collaboration quality after making a mistake. While previous research in HRI suggests that polite behavior can help mitigate the negative effects of robot mistakes (Srinivasan and Takayama, 2016), our goal is to identify the specific contexts in which a robot’s polite behavior, whether in correct or erroneous situations, is perceived positively by humans.

To enhance the robustness and broadness of our findings, we include two types of non-humanoid robots (a mobile robot and a manipulator) in two different task contexts. This allows us to assess how politeness strategies are perceived across various robot forms and interaction scenarios. Prior work highlights the importance of such replication across robot types to uncover behavioral patterns and to identify moderating variables—such as robot morphology or task characteristics—that may influence user perceptions of politeness and error (Ullman et al., 2021; Olatunji et al., 2021).

Thus, our study aims to address three main questions:

RQ1: Does robot politeness affect the perceptions of the robot by its users?
RQ2: Can robot politeness improve perceptions of a robot that errs?
RQ3: Do the answers to RQ1 and RQ2 depend on the context (e.g., different robots performing different tasks)?

The following section presents the theoretical background and conceptual framework of the study. Section 3 details the research methodology. Sections 4, 5 report the results and participants’ observations, which are discussed in Section 6. Conclusions are drawn in Section 7.

## 2 Theoretical framework and research background

### 2.1 Politeness

Studies exploring politeness in human-robot interaction (HRI) have produced mixed findings. Research has focused on studies on humanoid robots (receptionist robot (Salem et al., 2013; Salem et al., 2014); healthcare service (Lee et al., 2017); medication reminder robot (Zhu and Kaber, 2012); an adaptive expressive robot (Ritschel et al. (2019); and in animation (gate-keeping robot (Inbar and Meyer, 2019). Most of these studies were based on Brown and Levinson’s politeness theory (Brown et al., 1987) and its core concepts of *face* and *face-saving*. In interaction design, politeness strategies may include joking, apologizing, and taking blame, among other approaches used to mitigate perceived threats (Pemberton, 2011). However, we argue that this framework may not be best suited for studying all types of robots interacting with humans. First, the concept of *face* mainly addresses verbal communication, whereas interactions in HRI frequently extend beyond purely verbal exchanges. Second, the notion of *face* is highly sensitive to cultural variations among interacting humans (Kasper, 1990; Mao, 1994). Third, the concept of *face* is not well-suited to interactions with non-humanoid robots, whose primary mode of interaction is through a graphical user interface.

Therefore, the current study adopts a research framework grounded in politeness theory as applied to human-computer interaction (HCI). The theoretical framework for the study of politeness in HCI was suggested by Bar-Or et al. (2022), based on Lakoff’s politeness theory Lakoff (1973). Tolmach Lakoff (1990) defined politeness as “a system of interpersonal relations designed to facilitate interaction by minimizing the potential for conflict and confrontation inherent in all human interchange” (p. 34). She suggests that to be polite, one must follow three sub-rules: 1) “Don’t impose” your perspective or will on the other person, 2) “Give options” while interacting with the other person, and 3) “Be friendly” while interacting with the other person. Lakoff’s theory addresses not only the linguistic aspects of polite communication but also its behavioral aspects, making it well-suited for interactions with systems that rely on speech, such as in various human-robot interactions (Bar-Or et al., 2022). Moreover, compared to Brown and Levinson’s framework, Lakoff’s model is relatively simpler to implement and operationalize.

Based on Lakoff’s politeness framework, (Bar-Or et al., 2022) examined politeness in interactive systems and found that polite behavior improves collaboration efficiency and user perceptions. Their study showed that politeness in HCI influences user’s enjoyment, satisfaction and trust. User enjoyment refers to a user’s perception of the enjoyment derived from the interaction and serves as an indicator of the user’s willingness to engage with the robot over time (Heerink et al., 2008). User satisfaction is widely used as an indicator of the effectiveness of the system in the model proposed by DeLone and McLean (2003). In the context of human–robot interaction, communication behaviors such as politeness can influence users’ evaluation of the interaction, thereby affecting their overall satisfaction with the robot. Trust is defined as the capability of the trustee to perform a significant action to meet the trustor’s expectations and is known to enhance the latter’s reliance (Gurtman, 1992; Mayer et al., 1995). All HRI studies evaluating politeness were based on Brown and Levinson’s theory (Brown et al., 1987) and employed humanoid robots. Results show mixed findings. Some studies (Inbar and Meyer, 2019) showed that politeness had an impact on the perception, while others indicated no impact (Salem et al., 2013; Salem et al., 2014). For example, the humanoid robot in Salem et al. (2013), Salem et al. (2014) performed as a receptionist and incorporated two tasks: a chit-chat task and a direction-giving task. The results revealed that users had a positive perception of politeness in both tasks. However, politeness did not have a significant impact on HRI performance in either task. The adaptive expressive companion robot (Ritschel et al., 2019) evoked mixed gender-based responses from participants; men generally responded positively to polite robot behavior, whereas women tended to perceive polite behavior less favorably. A series of studies (Inbar and Meyer, 2019), investigated the role of politeness in a gatekeeper robot, by presenting participants with video clips of human-robot interactions. The results indicated that, irrespective of demographic differences, participants consistently preferred the robots that exhibited polite behavior (Inbar and Meyer, 2019). In a study involving a humanoid healthcare service robot, participants preferred interactions that combined polite gestures with direct commands over those featuring no gestures and indirect, albeit polite commands (Lee et al., 2017). Zhu and Kaber in (Zhu and Kaber, 2012) implemented a full politeness strategy based on Brown and Levinson’s theory to remind participants to take their medication while they were engaged in a primary task. Their findings revealed that a combination of positive and negative politeness strategies was particularly effective. A different study investigated the role of impolite behavior defined as “face-threatening behavior,” such as negative remarks that could impact the social image, during an exercise task involving a humanoid robot (Rea et al., 2021). While the impolite behavior led to improved participant performance, likely due to its compelling and competitive nature, participants did not favor the impolite robot.

The current study builds upon previous work (Kumar et al., 2021; Kumar et al., 2022b), which developed three levels of politeness for HRI using Lakoff’s rules: the No-rules level, where none of the rules are applied; the single-rule polite level, where only the “do not impose” rule was used; and the three-rules polite level, where all three rules were applied. The results indicated that higher levels of polite behavior during interaction led to greater user enjoyment, satisfaction, and trust in the robot, independent of gender (Kumar et al., 2021; Kumar et al., 2022b). However, the findings also suggested that participants were not sensitive to distinctions between the two polite levels (single-rule and three-rule conditions); they could only distinguish between polite (single-rule or three-rules) and non-polite (no politeness rule) behaviors. Drawing on these findings (Kumar et al., 2021; Kumar et al., 2022b), the current study employed two levels of politeness, namely, ‘polite’ and ‘No-rules polite’. In the ‘polite’ condition, all three sub-rules of Lakoff’s theory were applied; the robot greeted the participants at the beginning of the interaction (being ‘friendly’), offered them choices, and avoided imposing actions. In contrast, in the No-rules polite condition, none of these sub-rules were implemented; the robot imposed its actions or views, provided no options, and did not attempt to be friendly. It is important to clarify that in this condition, the robot’s behavior is closer to being “strict” rather than “impolite” in the sense used in Rea et al. (2021).

Given our focus on non-humanoid robots, we adopted Lakoff’s politeness framework for implementation, for the reasons outlined at the beginning of this section. To examine how politeness moderates users’ perceptions of an imperfect robot, we incorporated an error element into the design, as discussed in the next section.

### 2.2 Robot errors

The second theme of our work addresses the effect of robot errors on the overall HRI assessment of robot politeness. As robots increasingly proliferate in our society, research must address, among other factors, erroneous interactions between humans and robots (Mirnig et al., 2017). Despite the common perception that robots perform error-free actions (Mirnig et al., 2017), the reality has shown that robots can err (Bruckenberger et al., 2013; Wright et al., 2019; Lee et al., 2010; Honig and Oron-Gilad, 2018; Aliasghari et al., 2023; Giorgi et al., 2022). Previous research (Bruckenberger et al., 2013) has revealed that robot errors significantly impact people’s future beliefs about the robot. Users exhibit reduced trust in the reliability of a robot that has made errors (Bruckenberger et al., 2013). A robot’s behavior in response to a failure can have several consequences: it may reduce task performance, diminish user trust, and negatively influence users’ perception of the robot (Honig and Oron-Gilad, 2018); ultimately affecting their willingness to interact with or use the robot again (Wright et al., 2019). Honig and Oron-Gilad’s comprehensive survey (Honig and Oron-Gilad, 2018) offers a taxonomy of HRI failures, including the many ways a robot can execute an incorrect action. In the present study, we focus on wrong-action errors, as they are both common and well documented (Gehle et al., 2015).

Effective human-system interaction depends on error-free system performance. In HCI, effectiveness is ‘defined as ‘the accuracy and completeness with which users achieve specified goals” (ISO, 1988). In HRI, effectiveness similarly refers to how well a human-robot team accomplishes its goals, typically assessed by the extend to which tasks are successfully completed and errors avoided (Weiss et al., 2014).

Numerous studies have examined how erroneous robot behavior influences user perceptions (Mirnig et al., 2017; Bruckenberger et al., 2013; Salem et al., 2015; Ragni et al., 2016). Several of these studies found that faulty robots were perceived as less reliable and less trustworthy than faultless ones (e.g., (Salem et al., 2015; Ragni et al., 2016; Gideoni et al., 2022)). In the first of these studies, however, objective task performance (e.g., a person following the robot) was not affected by the robot’s errors (Salem et al., 2015). However, subsequent research reported that erroneous robots were perceived as less competent, intelligent, and reliable than error-free robots (Ragni et al., 2016) and that personalized failures negatively impact trust (Gideoni et al., 2022). Other findings present a more nuanced picture: when errors were unrelated to the task, participants perceived the faulty robot as more likable and not less intelligent than the perfectly performing robot (Mirnig et al., 2017). Similarly, (Flook et al., 2019) observed that participants sometimes perceived erroneous robots more positively than correct ones. However, in a service context, robot errors substantially lowered perceived service quality (Lee et al., 2010). A study involving a robot conducting medical checkups for suspected COVID-19 patients further showed that errors primarily undermined user trust and that trust was modulated by the level of transparency (Nesset et al., 2021). Finally, (Giorgi et al., 2022) found that although older adults preferred friendly robots, such friendliness did not offset the negative impact of erroneous behavior.

Different politeness strategies have been employed to mitigate the effect of errors. A study examining how robots mitigated malfunctions using three different strategies (positive polite strategy, negative polite strategy, and no strategy) (Lee et al., 2011), found that participants responded more positively when the robot acknowledged its mistake using a positive polite strategy. Conversely, another study (Engelhardt et al., 2017), reported that a robot that ignored errors was perceived more positively than one that politely acknowledged or explained them. Politeness has also been explored as a recovery mechanism following erroneous behavior, with findings showing that polite responses can restore users’ positive perceptions of the robot (Cahya and Giuliani, 2021). Related research in AI (Mahmood et al., 2022), further suggests that adopting a polite strategy of owning up to mistakes can mitigate the negative impact of errors. Similarly, adopting an “apology” strategy during interaction has been perceived as sincere (Ge et al., 2019), and apology-based error messages, viewed as a form of politeness, are generally received more positively by users (Yuan et al., 2020). When a robot both acknowledges its mistake and issues an apology, it is perceived as more likable, intelligent, and effective in mitigating errors (Hu et al., 2022). A recent systematic review further emphasizes the central role of politeness in HRI, particularly in resolving errors (Ribino, 2023).

Most prior studies have conceptualized politeness as a *reactive* mitigation tactic—such as apologizing used **after a robot fails**. However, the broader context of **robots that consistently behave politely yet occasionally make mistakes** remains largely unexplored. Earlier work Kumar et al. (2022a) has begun addressing this gap by examining how culture and gender influence responses to polite, error-prone robots, focusing on older adults—an important non-technical user group (Seibt et al., 2021; Veling and McGinn, 2021; Van Maris et al., 2020).

The present study advances this line of inquiry in two ways:

1. Robot diversity: We test both a mobile robot and a manipulator, enabling an assessment of politeness and error effects across distinct non-humanoid platforms performing different tasks.
2. Participant cohort: We focus on young adults with an engineering background to ensure a homogeneous participant group and to complement our previous studies involving older adults.

Through the systematic manipulation of politeness levels and controlled errors across two robot types, this study aims to evaluate the joint influence of polite behavior and robot correctness on user perceptions in everyday HRI.

### 2.3 Robot types and task scenarios

The HRI taxonomy has been categorized into three groups, i.e., interaction context, robot, and team classifications Onnasch and Roesler (2021). *The interaction context is clustered according to the field of application* (such as industry, service, military) and the type of interaction with the robot (such as field, laboratory). The robot could be classified according to task (e.g., manipulation, transportation), morphology (e.g., appearance, communication), and level of autonomy. Team classification involves the role of the human (supervisor, operator), team composition (equal or unequal contribution of human and robot), information about the communication channel (e.g., electronic, visual), and proximity (temporal, physical).

In this work, we implemented multiple levels of politeness across different robot types performing different tasks. Our previous research (Kumar et al., 2022b) highlighted that both robot type and task characteristics significantly influence how users evaluate a design. The results indicate that user satisfaction was significantly influenced by the type of robot (Kumar et al., 2022b). However, many HRI studies rely on brief interactions involving a single-robot or a single-task, an approach that limits the broadness of findings.

To address this limitation, we designed our study to include two types of non-humanoid robots—a mobile robot and a manipulator—each engaged in a different task. This design not only broadens the scope of the findings but also enables the identification of potential moderating variables or boundary conditions that shape the effects of politeness and robot correctness. By examining these effects across different settings, we aim to identify common trends that generalize beyond a single interaction context.

Although prior research has compared different robot types (Fong et al., 2003), such comparisons have typically focused on the robot’s physical form or anatomy (Sim and Loo, 2015; Bartneck et al., 2009; Rothstein et al., 2020; Yogeeswaran et al., 2016). Some work has also suggested incorporating different robots or tasks to better evaluate interaction design (Olatunji et al., 2021). A study (Kumar et al., 2022b), which tested three politeness levels, found that participants reported greater satisfaction when interacting with a mobile robot compared to a manipulator. Therefore, to enhance the broadness of the findings and further investigate the interplay between politeness, error, and robot context, the present study was intentionally designed to include multiple robot types and task scenarios.

### 2.4 Hypothesis

Implementation of Lakoff’s rules of politeness is expected to enhance users’ perceptions of the robot (Bar-Or et al., 2022; Kumar et al., 2022b). We posit that implementing all three politeness rules would positively influence key user perceptions during the interaction, including user enjoyment (Heerink et al., 2008), user satisfaction (DeLone and McLean, 2003), and trust (Gurtman, 1992; Mayer et al., 1995). Prior work suggests that such perceptions can be influenced by politeness strategies in HCI (Bar-Or et al., 2022) and HRI (Kumar et al., 2022b). We further expect these effects to hold irrespective of robot type, particularly in scenarios where the interaction proceeds correctly without errors.

*H1* Politeness will enhance user enjoyment, satisfaction, and trust during interaction, irrespective of robot type, in scenarios where the interaction proceeds correctly.

Politeness has also been explored as a strategy for mitigating the negative impact of errors in human–robot interaction (Lee et al., 2011). Extending this idea to polite robots, we posit that implementing politeness rules while the robot makes an error will help maintain positive user perceptions. Specifically, we expect that polite behavior during error situations will not negatively affect user enjoyment (Heerink et al., 2008), satisfaction (DeLone and McLean, 2003), and trust (Gurtman, 1992; Mayer et al., 1995) when compared to interactions without politeness rules. We further expect these effects to hold irrespective of robot type.

*H2* A polite robot making an error will not negatively impact user enjoyment, satisfaction, and trust during interaction, irrespective of robot type.

## 3 Methods

### 3.1 Study design

We conducted two user studies (Manipulator and cube arrangement task and Mobile robot and maze navigation task) to investigate the joint effects of a robot’s politeness and erroneous behavior rather than examining them in isolation on human-robot interaction (HRI). Each study involved a different type of non-humanoid robot, each assigned a distinct task: a manipulator robot engaged in a cube arrangement game and a mobile robot responsible for navigating a maze. In both studies, participants took on the role of supervisor, overseeing the robot’s performance.

Both studies were conducted with participants from a similar demographic background consisting of engineering students who are Hebrew speakers. As politeness is culturally situated, the experiments were intentionally conducted within a single linguistic and cultural context to explore the effectiveness and perception of politeness strategies within a specific cultural group. A within-subjects design was employed: for each robot-task pair, a different group of participants interacted with the robot under four experimental conditions defined by two independent variables: **politeness** (Polite vs. No-rules polite) and **operational correctness** (Correct vs. Erroneous). Politeness was operationalized using Lakoff’s three politeness principles: *Do not impose*, *Give options*, and *Be friendly*. Two levels of politeness were used: In the Polite condition, all three rules were applied; in the No-rules polite condition, none were used. The task-specific dialogues reflecting these politeness levels are provided in Tables 1, 2.

**TABLE 1:** The dialogue (in bold letters) that appeared on the GUI when participants interacted with the robot. In the polite condition, there were four different screens: the Start screen, Task screen, Instruction screen, and Finish screen. In the “No -rules” polite conditions, there were only two screens: the Start screen and the Finish screen.

GUI screen	Start screen	Task screen	Instruction screen	Finish screen
No-rules polite condition	“Arrange the cubes in the following manner.” (imposing actions and without giving options)			“Finish” not friendly salutation
Polite condition	“Shalom! my name is DOBOT, and I’m hereto help you with the game. Click next to proceed.” (Being friendly and not imposing views)	Left side of screen: (Figure 2): “Click on the below colored button to bring the respective colored cubes. “Below this instruction: Colored buttons representing colored cubes right side of window (Figure 2): “Arrange the cubes in the following manner.” (Giving options and not imposing views)	After clicking any colored button: “Arrange the cubes in the following manner.”	“Thank you very much.” (Being friendly)

**TABLE 2:** Dialogue (in bold letters) that appeared on the GUI during participant interaction with the mobile robot. In the polite condition, there were five different screens (Start, Task, Instruction, Test, and Finish screens, respectively). In the No-rules polite conditions, there were only three screens (Start, Test, and Finish screens).

GUI screen	Start screen	Task screen	Instruction screen	Test screen	Finish screen
“No- rules” polite condition	Instruction 1 “Move to the blue/red/green room” (depending on the target room) Instruction 2 “The letter must be inserted below.” Spaces were provided for placing the three letters			“Click on the test button to check the answer.”	“The answer is correct/wrong.” (depending on the user’s answer)
Polite Condition	“Shalom! My name is Waffle and I’m hereto help you with the game Click Next to proceed.” — *Being* *friendly*	Instruction 1 “Select the desired rooms.” Three buttons named “green”, “red”, and “blue” were shown— *Giving* *option* Instruction 2 “The letter must be inserted below.”	After clicking any button “Moving to blue/red/green” (depending on the selection)	“Please click on the test button to check the answer.”	“The answer is correct/wrong.” (depending on the user’s answer)

Robot behavior—either correct or erroneous—was implemented based on the nature of the assigned task for each robot. This yielded four distinct experimental conditions: No-rules polite and Erroneous (NPE), No-rules polite and Correct (NPC), Polite and Erroneous (PE), and Polite and Correct (PC). These combinations allowed us to systematically assess the effects of politeness and operational correctness, both independently and interactively, on user perceptions in HRI.

Each robot was paired with a specific task to create two distinct interaction contexts. The conditions are different and cannot be identical. Thus, the constructs were evaluated in different settings and interactions. This design enabled us to assess how the interplay between politeness and error varies depending on the type of robot and the nature of the task, thereby capturing a broader range of user experiences.

### 3.2 Experimental setup

#### 3.2.1 Manipulator and cube arrangement task

**FIGURE 1:**
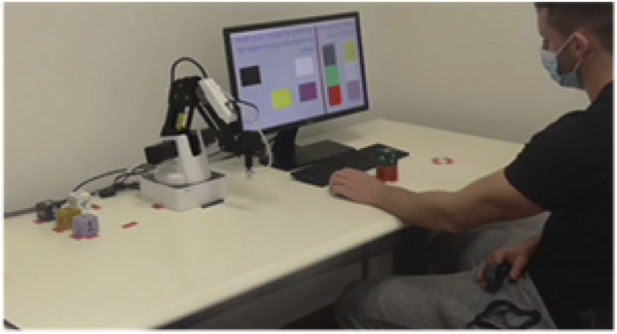
Participant interacting with the manipulator through the GUI. The participants interact with the GUI, and the robot’s task is to bring cubes near to the participants.

The task was to fulfill a cube sorting task with a 4-DOF DOBOT robot via a graphical user interface (GUI) implemented in Python (Figure 1). The participants were asked to arrange a set of colored cubes vertically according to the instructions given on the GUI (Figure 1). They received explanations before the experiment about the positions where each cube was to be placed, in the arrangement shown on the right side of Figure 1. When the robot brought the cube to a preset position, the participants had to select the cube and place it in the designated position.

At the start of the experiment, four different buttons were presented on the GUI named “Left,” “Left Middle,” “Right Middle,” and “Right”. The four buttons operated the conditions NPE, NPC, PE, and PC, respectively, but the participants were informed that they could choose the buttons in any order (they were not informed which button corresponded to which condition).

**FIGURE 2:**
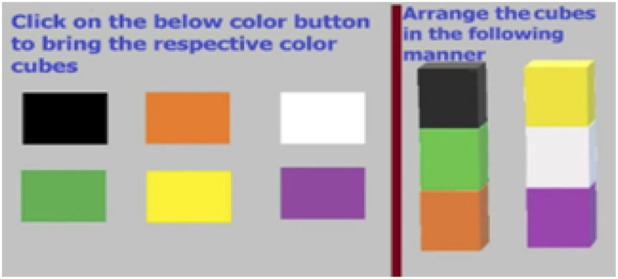
The task screens. Using the GUI, the user chose the specifically colored cubes. The colored buttons are located on the left side, and the user selects one in a way that causes the robot to bring the corresponding colored cubes. The instructions for arranging the cubes are on the right.

Human interaction with the robot included four distinct stages (Start, Task, Instruction, Finish), during which the robot’s GUI sent messages to the human operator. The dialogue of the polite condition and No-rules polite condition for the manipulator robot is shown in Table 1. At the Polite level, there were four different screens: the Start screen, the Task screen, the Instruction screen, and the Finish screen. In the Start screen, the robot first greeted the user (adhering to the rule of *Be friendly* politeness) (Table 1). Participants were supposed to click the “Next” button to proceed (following the rule *Don’t impose*). After clicking the next button, the Task screen appeared (as shown in Figure 2). The user now had the option of choosing colored buttons corresponding to the different colored cubes (adhering to the *Give. options* and *Don’t impose* rules) (Table 1). When the participants clicked on each colored button, the robot would bring the corresponding colored cube, and the Instruction screen would appear (for dialogue, see Table 1). The Instruction screen was comprised of two stacks of colored cubes that needed to be sorted (Table 1). After the robot brought one cube, the Task screen would reappear with the previously chosen colored button missing. At the end of the experiment, the Finish screen appeared with the dialogue shown in Table 1.

Under the No-rules polite conditions, the interaction with the robot consisted of only two distinct steps, each with its own screen: the Start and Finish screens. The Start screen appeared with instructions on how to arrange the cube. The robot brought the colored cubes one by one while the Start screen was displayed in the GUI. The participants were presented with a screen display of colored cubes and were requested to arrange the colored cubes according to the instructions displayed on the GUI. The dialogue of the Start screen for the No rule polite condition is shown in Table 1. After the robot had brought all the cubes, the Finish screen appeared (Table 1). In the No-rules polite condition, the robot did not exhibit any of the polite behaviors (details in Table 1).

In the correct level of the No-rules polite robot, the robot presented the colored cubes in an order that would be easy for the user to arrange. For example, the robot would bring the orange cube, the green cube, and then the black cube. The instructions for arranging the cubes are shown on the right side of Figure 2. In this case, it was easy for participants to arrange the cubes. In the erroneous behavior of the No-rules polite robot, the robot did not present the colored cubes in the order required for the arrangement. For the same example, the robot would bring the orange cube, and instead of bringing the green cube, it would bring the black cube, in contradiction to the expected order.

In the erroneous behavior condition of the Polite robot, participants clicked the colored button, as shown in Figure 2, and the robot would bring a wrong-colored cube. For example, if the participants clicked the black button on the GUI (Figure 2), the robot would bring a red colored cube. In the correct condition of the polite robot, the robot would bring the correct colored cube upon the click of the colored button on the GUI (Figure 2).

#### 3.2.2 Mobile robot and maze navigation task

**FIGURE 3:**
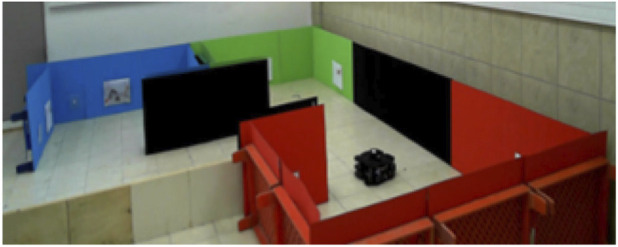
The environment in which the mobile robot tours the rooms. Each room is colored in a different color, and contains one letter of a three-letter word.

**FIGURE 4:**
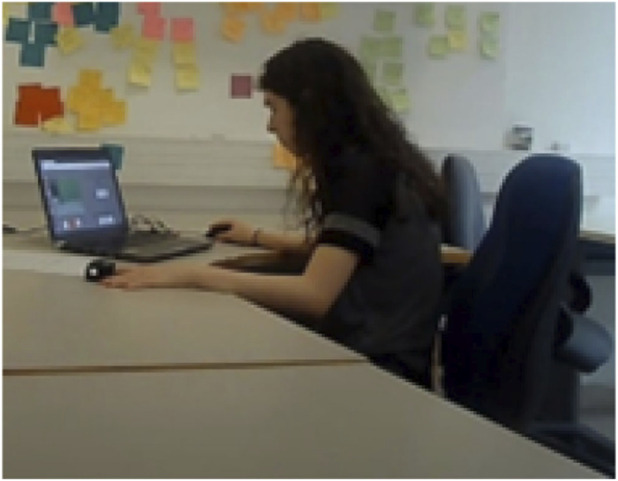
Participant interacting with the GUI of the robot. Participants were seated so that they did not see the robot. The GUI contains live camera feedback from the robot.

The task in this experiment was to put together a word consisting of three letters that were scattered in a defined environment—in our case, in three different rooms in a maze setup (Figure 3). The users were instructed to remotely navigate a mobile robot (a Turtlebot Waffle) to the three rooms. The interaction between the participants and the robot was facilitated through a GUI (Figure 4). The dialogue of No-rules polite and polite conditions behavior is shown in Table 2.

During the task, the user did not see the environment directly but rather through a camera installed on the robot. The live video taken from this camera was displayed on the GUI. The experiment included four conditions (trials) in which the user was required to compose four different three-letter words, all belonging to the same category, e.g., animals. In each condition, a different word was selected. Before the experiment started, the user was informed of the order in which the robot would visit the rooms (the order was identical in all trials). This experiment included the following six rules: 1) Each word consisted of three different letters (i.e., there were no duplicate letters); sample words were the Hebrew words for Dog, Sheep, Goat, and Camel (all consisting of three letters). 2) The user was unaware of the letters and the words before the start of each trial. 3) Each of the three letters was stored in a different room, with the rooms being named Blue, Red, and Green. 4) To complete the task efficiently, the robot was required to visit each room in the correct order, which was displayed to the user *a priori*; namely, the participant was informed about the correct order of the rooms but not about the word to be completed. 5) In each condition, the robot visited each room only once. 6) Finally, based on the letters revealed to the user, the user composed the three-letter word. The sequence of the specific conditions (PC-PE-NPC-NPE) was randomly selected (to ensure that there was no order effect) and then assigned to the participants.

**FIGURE 5:**
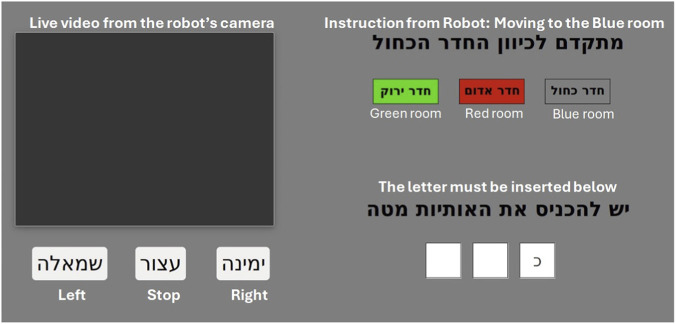
GUI designed for the mobile robot. The text of the experiment was in Hebrew, and translations are shown in white. A live picture from the robot’s camera is displayed on the left side of the GUI. The three buttons below the screen (left, right, and stop) are used to teleoperate the robot. On the right are three buttons representing the three rooms and three boxes where the participant would record the letters.

In the Polite condition, the interaction with the robot involved five stages associated with five screens (Start, Task, Instruction, Test and Finish), during which the robot sent the appropriate messages through the GUI screen as shown in Table 2. The start screen appeared at the start of each Polite level with the robot greeting the participants (“Be friendly”) at the start of the experiment, as demonstrated in Table 2. In the next screen, the robot provided users with the option (i.e., each colored button representing a room) to help them navigate to a room (“Don’t impose” and “Give options”), as demonstrated in Table 2. The GUI of this screen is displayed in Figure 5. When the participants clicked one of the colored buttons, the robot moved to a particular room. Simultaneously, instructions appeared on the instruction screen (see Table 2). After the robot navigated to the chosen room, the Task screen reappeared, but the button for the rooms already visited was missing. At the end of the condition, the Test screen (see Table 2) appeared. After the participants filled the boxes with letters and clicked the “test” button, the Finish screen appeared (Table 2).

In the No-rules polite condition, there were three screens, namely, the Start screen, the Test screen, and the Finish screen (Table 2). At the start of the experiment, there were two instructions, as shown in Table 2. The first instruction told the user to navigate to a particular room, and the second instruction was related to filling in the letters of the word. The GUI was similar to Figure 5 except for the buttons showing a different room. Once the experiment began, the robot proceeded to one room and waited for 1 minute, allowing participants to note down the letter. Afterwards, the robot moved to the next room. After the robot had navigated to all of the rooms and the participants had filled in the letters, a test screen appeared. The instruction is shown in Table 2. After checking the letters chosen, the end screen appeared with instructions as shown in Table 2.

In the correct condition, the robot with No-rules navigated between the rooms according to the correct letter sequence for assembling the word. For example, for the word “cat”: the letters, “c”, “a”, and “t” are in the blue, green, and red rooms, respectively. In this condition, the robot navigated to the blue room, the green room, and the red room in this order. In the NPE condition, the robot navigated to each room, but not in the correct order. In the PE conditions, the user would press a colored button to access a particular room, but the robot would go to the room with a different color. In the above example, if the participant pressed the green button, the robot navigated to the red room.

### 3.3 Participants

Thirty engineering students participated in each user study (Table 3). All participants were recruited from the third-year Industrial Engineering program and had prior exposure to robotic systems through coursework that included hands-on interaction with robots and GUI-based tools. Participants were therefore assumed to possess sufficient digital literacy and familiarity with such systems. The sample was relatively homogeneous, with all participants being Hebrew speakers and sharing a similar cultural background. The two studies involved separate participant groups. Due to a technical malfunction in the robot operated by one male participant, the mobile robot study analyses included data from 29 participants (aged 23–35). The manipulator cube study included 30 students aged 24-28. Participants were recruited via a course website, and volunteers received one course credit point credit as compensation for participation.

**TABLE 3:** Gender and age distribution of the participants in each experiment.

Experimental description	No. of males	Age	No. of females	Age
Manipulator robot	15	Range: 25–28 Mean = 26.47 (0.99)	15	Range: 24–2 Mean = 25.4 (0.91)
Mobile robot	14	Range: 25–31 mean = 27 (1.52)	15	Range: 23–35 Mean = 25.67 (2.92)

We deliberately recruited a demographically homogeneous group with similar age and experience to minimize potential confounding factors. This approach enabled clearer isolation of the effects of our HRI manipulations—politeness and error—by reducing variability and novelty effects commonly observed among inexperienced users (Ullman et al., 2021).

### 3.4 Experimental procedure and measures

At the start of each session, participants received written instructions outlining the robot’s role and the task requirements.

- Manipulator-robot task: Participants learned that the robot would deliver colored cubes on request, and that they would use the on-screen interface to arrange those cubes according to a specified pattern.
- Mobile-robot task: Participants were told that the robot would autonomously visit three rooms. In each room, it would collect letter tiles, which the participants would then arrange to form a target word.

On purpose, participants were not told anything about the robot’s politeness conditions; the politeness level was embedded covertly within the interaction. After signing the consent form, they completed a preliminary questionnaire regarding their age, gender, and educational background.

User perceptions were assessed through three key measures—**enjoyment**, **satisfaction**, **trust** - using structured questionnaires. Additionally, **preference** was evaluated using post-experiment questions, following the procedures adopted in our previous study Kumar et al. (2022a).

Although additional dimensions, such as frustration and qualitative feedback (e.g., open-ended responses to the experimenter) were observed, they fall outside the scope of this study and will be addressed in future research.

We employed a within-subjects design. After completing each condition, participants completed a brief post-trial questionnaire (Table 4) assessing their perceptions of the robot in terms of enjoyment, satisfaction, and trust. Each measure was measured using two items rated on five-point Likert scales (1 = “strongly disagree,” 5 = “strongly agree”). The two items corresponding to each measure were averaged to obtain a composite score. Questionnaires were administered immediately after each condition to capture interaction-specific perceptions and minimize recall bias.

**TABLE 4:** Post trial questionnaire for each measure. Each measure has more than two questions. The response to each question was a Likert scale rating from 1 to 5.

Measures	Questions	References
Satisfaction	(1) Interacting with the robot was a pleasant and satisfactory experience	Kumar et al. (2022b), Kumar et al. (2022a)
	(2) I was satisfied with the experience of using a dialogue with the robot tocomplete tasks	
Trust	(1) The robot was trustworthy	Lee and Choi (2017); Kumar et al. (2022a)
	(2) I can trust the information provided by the robot	
Enjoyment	(1) It was enjoyable to sharea conversation with the robot	Kumar et al. (2022b), Kumar et al. (2022a)
	(2) The conversation with the robot was exciting	

After completing all four conditions (NPE, NPC, PE, and PC), participants completed a separate post-experiment questionnaire (Table 5). This questionnaire consisted of five questions (Table 5) designed to elicit comparative reflections across conditions. Question 1(a) asked participants in a binary Yes/No format, whether they perceived differences between the scenarios. Participants who responded affirmatively were asked to describe the perceived differences in Question 1(b), an open-ended short-answer question. The remaining questions (Questions 2–5) were multiple-choice items in which participants selected one of the four conditions as their response. These questions asked participants to indicate which condition they considered best for performing the task, least effective for performing the task, most polite, and least polite. Unlike the post-trial questionnaires, which measured condition-specific perceptions, the post-experiment questionnaire served a comparative and reflective purpose across all conditions. Participants were invited to provide brief written comments describing their experiences and the differences they perceived across the interaction conditions. These responses were collected as part of the questionnaire rather than through interviews or think-aloud protocols. No audio recordings were made, and no formal qualitative coding procedures were applied. No additional interviews or open-ended qualitative data collection procedures were conducted. The summary of the questionnaire is presented in Table 6.

**TABLE 5:** Post experiment questionnaire.

(1) (a) Did you feel the differences between the scenarios?
(b) If so, what was the difference?
(2) Which scenario is better for performing this task?
(3) Which scenario is the least good for performing this task?
(4) In which scenario would you say that the robot was the most polite?
(5) In which scenario would you say that the robot was the least polite?

**TABLE 6:** Summary of measurement instruments by robot type.

Robot type	Sample size (N)	Instrument type	Timing of measurement	No of items	Measures	Debriefing/Interview
Manipulator robot	30	Questionnaire (likert-scale)	End of each condition	3	Enjoyment, satisfaction, trust (Table 4)	No
		Post experiment question	After experiment	5	(Table 5)	
Mobile robot	29	Questionnaire (likert-scale)	End of each condition	3	Enjoyment, satisfaction, trust (Table 4)	No
		Post experiment question	After experiment	5	(Table 5)	

### 3.5 Analysis

A Kolmogorov-Smirnov test indicated that most data were not normally distributed (Table 7). Therefore, the Friedman Test was applied to evaluate the influence of the robot condition (NPC, NPE, PC, and PE). A post-hoc Dunn test was conducted when a significant difference was found among the independent variables in the Friedman test. The findings for each measure are presented with descriptive (median η ) and statistical test analysis. As mentioned earlier, the order of robot conditions was either randomized (for the manipulator robot) or chosen by the participant (for the mobile robot). The significance level for all statistical tests was set at 5%, with a Bonferroni-corrected alpha of 0.0167 to account for multiple comparisons.

**TABLE 7:** Summary of Kolmogorov-Smirnov test results. Enjoyment and Satisfaction for the manipulator robot were the only measures that displayed a normal distribution. All other measures were not normally distributed.

Robot type	Enjoyment	Satisfaction	Trust
Manipulator robot	D = 0.11007 p = 0.1092	D = 0.10703 p = 0.1279	D = 0.18349 p** <0.001
Mobile robot	D = 0.20127 p** <0.001	D = 0.14759 p** <0.001	D = 0.21801 p** <0.001

## 4 Results

The analyses and results are organized by dependent variable. For each variable, we first present descriptive statistics, followed by the results of statistical tests assessing the effects of the experimental condition for each robot/task type. Separate analyses were conducted for all three dependent variables: enjoyment, satisfaction, and trust. Notably, all participants successfully completed the full set of tasks in both experiments.

The measures of enjoyment, satisfaction, and trust showed good internal consistency. Both the enjoyment and satisfaction measures showed good reliability (standardized Cronbach’s α = 0.86, 2 items; 95% CI [0.79, 0.91]), standardized Cronbach’s α = 0.85, 2 items; 95% CI [0.79, 0.90]). The trust measure demonstrated excellent reliability (standardized Cronbach’s α = 0.98, 2 items; 95% CI [0.96, 0.99]).

### 4.1 Robot behavior

**FIGURE 6:**
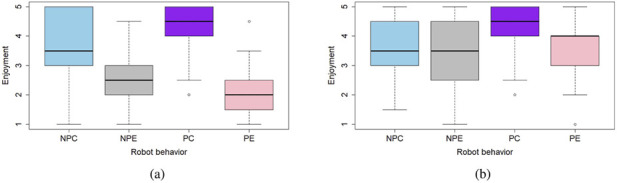
Box plot of the enjoyment measure for the experiment with the manipulator and the mobile robot. **(a)** Manipulator robot **(b)** Mobile robot.

**FIGURE 7:**
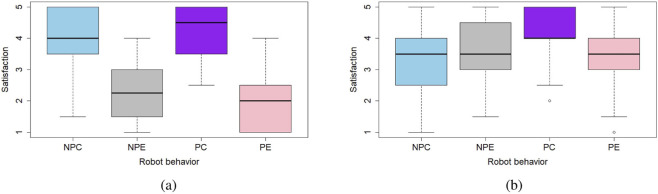
Box plot of the satisfaction measure for the experiment with the manipulator and the mobile robot. **(a)** Manipulator robot **(b)** Mobile robot.

**FIGURE 8:**
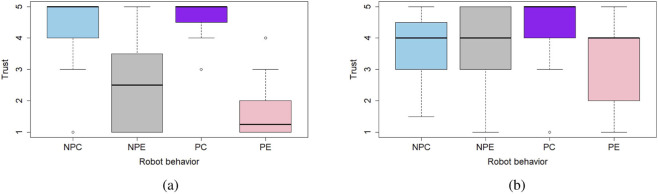
Box plot of the trust measure for the experiment with the manipulator and the mobile robot. **(a)** Manipulator robot **(b)** Mobile robot.

In both tasks/robot types, participants gave better ratings for the PC condition than for all other conditions. On average, they enjoyed interacting with the PC robot more (Figures 6a,b), were more satisfied during the interaction with it (Figures 7a,b), and trusted it more (Figures 8a,b) than the robots in all the other conditions. The Friedman test revealed significant differences between the experimental conditions for all three independent measures: enjoyment $\left(\chi^{2}\left(3\right)=59.21,p<0.001\right)$ ; satisfaction $\left(\chi^{2}\left(3\right)=72.51,p<0.001\right)$ ; and trust $\left(\chi^{2}\left(3\right)=70.72,p<0.001\right)$ . The medians are reported in Table 8. Furthermore, the post-hoc Dunn test (Table 9) revealed that, for all dependent variables, the PC condition was significantly different from the NPE (except trust for the mobile robot, which was marginally significant) and PE for all types of robots/tasks. It was also significantly different from the NPC in the mobile robot experiment. In addition, NPC was significantly different from PE and NPE for the manipulator robot.

**TABLE 8:** Median of the different measures broken down by robot type and experimental condition.

Measures	Enjoyment		Satisfaction		Trust	
Robot type	Manipulator robot	Mobile robot	Manipulator robot	Mobile robot	Manipulator robot	Mobile robot
NPE	η = 2.50	η = 3.50	η = 2.25	η = 3.50	η = 2.50	η = 4.00
NPC	η = 3.50	η = 3.50	η = 4.00	η = 3.50	η = 5.00	η = 4.00
PC	η = 4.50	η = 4.50	η = 4.50	η = 4.00	η = 5.00	η = 5.00
PE	η = 2.00	η = 4.00	η = 2.00	η = 3.50	η = 1.25	η = 4.00

**TABLE 9:** Summary of *post hoc* pairwise test results between the conditions for each measure for each of the robot types.

Scenario	Enjoyment		Satisfaction		Trust	
	Manipulator robot	Mobile robot	Manipulator robot	Mobile robot	Manipulator robot	Mobile robot
NPCvs.NPE	z = 4.22 p = <0.0001	z = 0.12 p = 1.00	z = 4.95 p = <0.0001	z = −0.14 p = 1.00	z = 4.85 p = <0.0001	z = −0.16 p = 1.0
NPCvs.PC	z = −1.29 p = 1.0	z = −2.95 p = 0.016	z = −0.67 p = 1.00	z = −3.28 p* = 0.006	z = −0.95 p = 0.4819	z = −2.62 p = 0.052
NPEvs.PC	z = −5.51 p = <0.0001	z = −3.06 p = 0.013	z = −5.62 p = <0.0001	z = −3.13 p = 0.01	z = −5.81 p = <0.0001	z = −2.72 p = 0.0833
NPCvs.PE	z = 4.98 p = <0.0001	z = 0.26 p = 1.00	z = 5.81p = <0.0001	z = 0.26 p = 1.0	z = 6.57 p = <0.0001	z = 1.42 p = 0.92
NPEvs.PE	z = 0.72 p = 0.8899	z = 0.14 p = 1.0	z = 0.87 p = 1.00	z = 0.40 p = 0.8097	z = 1.71, p = 0.52	z = 1.51, p = 0.67
PCvs.PE	z = 6.27 p = <0.0001	z = 3.21 p = 0.007	z = 6.49 p = <0.0001	z = 3.54 p = 0.002	z = 7.52 p = <0.0001	z = 4.04 p = 0.0003

### 4.2 Preferences

Based on responses from the post-experiment questionnaire (Table 5), we identified the most and least preferred conditions. All the participants (100%) were able to distinguish between the conditions. PC was the most preferred condition for both experimental groups (60% for the manipulator robot and 62% for the mobile robot).

The least preferred condition (Q3 Table 5) in the manipulator robot experiment was PE (57%), while in the mobile robot experiment, 41% of the participants indicated that the NPE was the least preferred condition. Additionally, all the participants noted that the PC was the politest condition (77% manipulator robot, 72% mobile robot). The least polite condition in the manipulator robot experiment was the NPE condition (50%) and PE (43%). In the mobile robot experiment, 31% of the participants noted that the NPC and NPE were the least polite conditions. Finally, all the experimental groups reported that the PC was the most enjoyable and the most satisfying condition, and in that condition, the participants were more confident that they had succeeded in the task.

## 5 Participant’s observations

The participant observations reported in this section are based on responses to the open-ended question (Question 1(b)) included in the post-experiment questionnaire (Table 5).

Participant 3 noted in the manipulator experiment that “In some scenarios [Polite], I was able to control which cube would be delivered to me and thus determine the order of the cubes, while in other scenarios, I had no such control [No-rules polite]—the robot did it on its own. Moreover, in scenarios where I could not predict the robot’s actions, I did not feel confident using it [Errorneous].”

Participant 14 noted in manipulator experiments that “In the right scenarios [Polite], the robot responded to my requests, compared to the left scenarios [No-rules polite] where it acted independently. In addition, on each side, there was a significant difference when the robot did what was expected of it versus when it did something else.”

Participant 32 noted in the mobile robot experiment that “Scenario 1 [PC] - It operates completely according to my choice (when you exit from room to room, it rotates). Scenario 2 [PE] - It changed between rooms - I pressed red, and it sent me to green. Scenario 3 [NPC] - It did not let me schedule the exits and went in its own room order, and there were no smileys, *etc.*, in the feedback. Scenario 4 [NPC] - It was in the correct room order, but continued from room to room immediately after I entered a letter.”

Participant 58 “In the first and second scenarios [NPC and NPE], the robot had most of the control over the situation. In the third and fourth scenarios [PE and PC], the room selection menu made it easy to work with the robot. The worst scenario [PE] for me is the third scenario because in this scenario, you lose trust in the robot.”

The observations reported in this section are derived from the questionnaire item that invited participants to describe the differences they perceived across the four interaction scenarios. Because the question was intentionally framed broadly, participants frequently described their experiences in terms of interaction outcomes such as control, predictability, responsiveness, and confidence in the robot’s behavior rather than explicitly referring to the specific politeness condition. Consequently, many responses emphasized aspects related to controllability, predictability, and reduced confidence following error occurrence. This pattern aligns with the tendency of users to evaluate interaction quality primarily at a functional level. Although the politeness manipulation was implemented according to Lakoff’s rules, participants typically expressed its effects indirectly through perceived changes in control and reliability during the interaction. Accordingly, the qualitative observations reflect participants’ interpretations of the overall interaction experience across the four scenarios, rather than direct commentary on the linguistic politeness itself.

## 6 Discussion

We implemented politeness strategies in two studies with different robot types, each performing a distinct task. This dual-robot approach addresses a common limitation in most HRI research - namely, the reliance on a single robot or task, which can constrain the findings. By incorporating two robot types and task contexts, our study facilitates the identification of consistent patterns, potential moderating variables, and boundary conditions that influence how robot correctness and politeness affect user perceptions. Evaluating these measures across different settings allowed us to uncover broader trends and enhance the applicability of our findings.

Given that real-world robots are inherently susceptible to errors, we specifically examined how users perceive a polite robot that makes mistakes. By investigating politeness and error together, rather than in isolation, we aimed to gain a deeper understanding of user experience and responses to complex, error-prone interactions with robots.

The interaction with the robot was mostly GUI based. While this setup may capture user responses to GUI-mediated phrasing during robot errors, we view this as an important first step toward understanding how politeness strategies can be implemented in non-humanoid robotic systems, where interaction often occurs through graphical interfaces rather than speech or anthropomorphic cues. The results of this study showed that correct robot behavior, regardless of politeness, was consistently associated with positive user perceptions (enjoyment, satisfaction, and trust) in the case of the manipulator robot. However, PC behavior was perceived more positively than other types of behavior in mobile robots (H1 is rejected).

Erroneous robot behavior led to negative user perceptions across both tasks. Importantly, politeness did not mitigate these negative effects, offering no significant improvement in how users perceived erroneous robots (RQ2) (H2 is rejected). Politeness had a modest positive effect on user perceptions of the **correct** mobile robot, but not on the correct manipulator robot (RQ1).

In contrast, for the manipulator robot, any condition involving correct behavior (PC or NPC) was perceived more positively than those involving errors, with politeness having no additional influence (RQ3). These findings highlight how the effects of politeness and correctness may be moderated by the robot type and task context.

An important overarching finding of this study is the **context-dependent nature** of how robot type, task, and behavior influence user perceptions. The effects were not uniform; rather, they varied depending on the interaction between these factors. This shows the importance of evaluating HRI under different conditions.

In the discussion below, we unpack both the main effects and their interactions, organized around the three experimental variables:

1. Correct vs. erroneous behavior
2. Polite vs. non-polite behavior
3. Robot/task type

### 6.1 Erroneous behavior

In both robot tasks, participants clearly distinguished between correct and erroneous robot behavior, consistently favoring the correct behavior. As illustrated in Figures 6a, b, 7a, participants reported greater enjoyment, higher satisfaction, and increased trust in robots that performed their tasks correctly compared to those that made errors. Contrary to the findings of Mirnig et al. (2017), user perceptions of the erring robot in this study were notably less positive—and in many cases, explicitly negative—compared to those of a robot that performed its tasks correctly.

It is important to note that the present study followed a within-subjects design, in which all participants experienced all four interaction conditions (NPE, NPC, PE, PC). Prior studies have often used between-subjects designs when examining erroneous robot behavior Mirnig et al. (2017). However, erroneous robot behavior is generally not preferred by users. In our results, the negative impact of errors varied across robot types and their associated tasks, with the effect being more pronounced for the manipulator robot than for the mobile robot. These differences in user perception are not unexpected and highlight the importance of evaluating HRI across diverse robot forms and task contexts. While we can only speculate about the cause of this variation, one plausible explanation is that participants were more visually and physically engaged with the **mobile robot’s movement**, which may have diverted their attention from the task outcome. In contrast, interactions with the **manipulator robot** occurred at close range, making its actions—and any errors—more immediate and noticeable. As a result, users may have been more sensitive to and affected by errors in that setting.

A second, complementary explanation may relate to participants’ domain knowledge, although this interpretation should be treated with caution. Since all participants were engineering students, they may have recognized that autonomous navigation is inherently more difficult than tabletop manipulation and therefore judged the mobile robot’s errors more leniently. However, we do not directly test the role of domain expertise in this study, and therefore cannot draw causal conclusions about its influence. Moreover, task affordances differed: in the mobile-robot scenario, participants did not control the robot’s movement and had no means to correct its mistakes, whereas in the manipulator-robot scenario, they could readily correct misplaced cubes. The ability to intervene—and the close-range visibility of each action—may have heightened their awareness of, and intolerance for, the manipulator’s errors, while making them more forgiving toward the mobile robot. These factors provide a more immediate explanation for the observed differences, independent of assumptions about participants’ technical background. These explanations are not mutually exclusive; several may operate simultaneously. Together, they highlight a broader methodological lesson: future HRI research should incorporate multiple robot types and task contexts to enhance generalizability and to pinpoint the task- and form-specific factors that shape user perceptions. T This aligns with the growing consensus that people respond differently to different robots and to the tasks those robots perform. For instance, Loi et al. Loi et al. (2018) showed that participants’ willingness to follow a robot’s instructions varied with the nature of the task.

When operating the manipulator, correctness affected the measures irrespective of politeness behavior. These results (Table 9) are similar to the previous study Kumar et al. (2022a). However, this finding contradicts previous research suggesting that the robot performance is of relatively low importance Salem et al. (2015). For the mobile robot, perceptions were negatively (but not strongly) affected by all conditions that are not both correct and polite.

For the manipulator robot, **operational correctness—regardless of politeness—drove user ratings** of enjoyment, satisfaction, and trust (Table 9), echoing earlier findings Kumar et al. (2022a). This result diverges from work by Salem et al. (2015), which argued that robot performance often plays a relatively minor role in user evaluations. In contrast, perceptions of the **mobile robot** declined whenever its behaviour was not simultaneously **correct *and* polite** (Figures 6b, 7b, 8b). Taken together, these patterns highlight the concept of the *error-cost*. When errors carry a **low cost**—and can be readily corrected by the user, as in the cube-arrangement task—mistakes have a smaller impact on user experience. When errors impose a **high cost** or are difficult to fix, users become less forgiving, and their overall assessment of the collaboration suffers.

### 6.2 Politeness

When interacting with the **correctly behaving manipulator robot**, **politeness did not significantly enhance participants’ perceptions** compared to the non-polite version, which already received high ratings (Table 9). This result aligns with previous findings Kumar et al. (2022a), which showed that participants had limited ability to distinguish between different levels of politeness. This finding is consistent with Giorgi et al. (2022), which reported that the robot’s friendliness did not mitigate the effects of its erroneous behavior. In contrast, **for the mobile robot**, where perceptions of the non-polite version were slightly lower to begin with, **politeness led to a noticeable improvement in user evaluations**. This finding is in line with earlier research demonstrating the positive effects of politeness in mobile robot interactions Kumar et al. (2022a); Inbar and Meyer (2019). For example, previous work Kumar et al. (2022b) showed that politeness significantly influenced user perceptions for both manipulator and mobile robots, with the highest level of politeness—defined by adherence to all three of Lakoff’s rules—being clearly distinguishable and evaluated more positively by participants. However, the current study only partially replicates these findings: while some politeness effects were observed with the mobile robot, no such effect emerged for the manipulator robot.

Rather than concluding that politeness has no impact in the case of the manipulator, a more plausible explanation is the **ceiling effect**—that is, participant ratings may have already reached a high level due to the robot’s correct and effective performance, leaving little room for politeness to further enhance perceptions. However, we note that this explanation is not directly tested in the present study, as we did not assess scale saturation or sensitivity. In contrast, the earlier study Kumar et al. (2022b) did not involve erroneous robot behavior, which likely allowed for greater variability in user perceptions, thereby enabling politeness to have a more pronounced influence on evaluations. Notably, the study revealed that polite behavior did not improve user perceptions when the robot made errors, regardless of the robot’s type. This finding aligns with prior research showing that politeness alone does not necessarily enhance user evaluations Lee et al. (2017). Nevertheless, these interpretations should be considered with caution, as the underlying mechanisms (e.g., expectation effects or scale limitations) were not directly measured. We initially expected that a polite robot committing an error would be perceived more favorably than a non-polite robot committing the same error. However, our results did not support this assumption. In fact, for the manipulator robot, participants reported *lower* levels of satisfaction and trust in the polite-erroneous (PE) condition than in the non-polite-erroneous (NPE) condition (Figures 6a, 8a).

Although we cannot offer a definitive explanation for this outcome, one plausible interpretation is that polite behavior raised user expectations regarding the robot’s competence or reliability. When these heightened expectations were subsequently violated by an error, the resulting mismatch may have contributed to more negative evaluations, consistent with the notion of a *disconfirmation* effect Oliver (1977). However, we note that this interpretation remains speculative, as the present study did not directly measure user expectations, perceived competence, or expectation–performance discrepancies. This interpretation can be further contextualized by contrasting politeness with other social robot behaviors. Prior work has shown that robot attentiveness can mitigate the negative impact of errors in human–robot interaction Manor et al. (2025). One possible distinction is that attentiveness operates as a dynamic and responsive signal during interaction, whereas politeness in our study was conveyed primarily through static textual cues. As such, politeness may increase expectations without providing adaptive feedback during error occurrences, potentially making users more sensitive to failures. Nevertheless, this account should be interpreted cautiously and warrants direct empirical investigation in future work.

### 6.3 Type of robot and task

The statistical analysis revealed that both robot type and task had a significant influence on user perceptions. Specifically, errors had a stronger negative impact on user perception in the case of the manipulator robot compared to the mobile robot. Additionally, while politeness had a modest positive effect on perceptions of the correct mobile robot, it had no noticeable impact on perceptions of the correct manipulator robot. As noted earlier, the explanations for these findings remain speculative and require further investigation in future studies.

One possible explanation is that in the mobile robot experiment, the cognitive load was a bit higher. In the polite condition, participants were given the option (via a button on the GUI) to direct the robot to a designated room—similar to the manipulator robot scenario—where they could instruct it to pick up a cube. In the mobile robot task, participants had to simultaneously interact with the robot and solve a word puzzle, increasing their cognitive load. In contrast, the manipulator task required participants to arrange colored cubes according to a visual layout displayed on the GUI. Another possible factor is the mode of interaction and physical proximity to the robot. In the manipulator task, participants were in close proximity to the robot and interacted with it via a GUI positioned near the robot, allowing for direct observation of its actions. In the mobile robot task, participants interacted through a GUI interface without direct visual contact with the robot itself, relying instead on a camera feed. This difference may have made it easier for participants to detect errors in the manipulator task and possibly heightened their expectations, which, when unmet, led to greater disconfirmation Oliver (1977) in the error conditions.

Taken together, these findings suggest that the effects of politeness are context-dependent and may be moderated by task characteristics and interaction conditions. In particular, when task demands or performance-related factors are more salient, they may overshadow the influence of politeness on user perceptions. These findings underscore the limitations of studying human-robot interaction through isolated, snapshot interactions that involve a single robot type or task. To the best of our knowledge, no prior studies have examined the effects of polite behavior in robots that make errors across two distinct types of non-humanoid robots. This emphasizes the importance of conducting future research that explores similar social dynamics across diverse robot forms and tasks to enhance the broadness and robustness of HRI findings.

### 6.4 Effect of politeness and erroneous behavior on trust

The current study operationalizes and evaluates trust using two-item measures. However, interpersonal trust is inherently multidimensional (Anzabi and Umemuro, 2023; Gompei and Umemuro, 2018) and comprises two key components: cognitive trust and affective trust. Cognitive trust is grounded in performance-based assessments and emerges from knowledge gained through interaction, which in turn leads individuals to trust the trustee with a definable level of confidence. While we did not measure these dimensions separately, this distinction provides a useful lens for interpreting our findings. Affective trust, in contrast, is grounded in emotional bonds that involve perceptions of care and concern. In the current study, the correct behavior of both robots primarily influences the cognitive dimension of trust, as incorrect behavior is perceived as the least trustworthy. These two components are intertwined and cannot be treated independently (Anzabi and Umemuro, 2023). This interdependence is reflected in the mobile robot condition, where only a marginal difference is observed between the PC and NPC conditions, with PC being slightly more favorable than NPC, PE, and NPE. However, as our study did not directly assess cognitive and affective trust, these interpretations remain speculative and should be validated in future work using multidimensional trust measures.

We employed non-anthropomorphic (non-humanoid) robots to evaluate the effects of politeness in a real-world scenario in which robots may commit errors. A previous study by Christoforakos et al. (2021), which employed a humanoid robot, demonstrated that the anthropomorphic nature of robot-generated cues does not directly influence trust. However, robots exhibiting warm or competent behavior are typically perceived as more anthropomorphic—and consequently more trustworthy—than robots displaying low warmth or incompetent behavior. In the present study, we found that combining polite behavior with correct task execution was associated with higher trustworthiness. In contrast, polite behavior did not mitigate the negative impact of erroneous behavior on trustworthiness. It is important to note that the use of non-humanoid robots, in combination with erroneous behavior, may have moderated the effect of politeness on perceived trustworthiness, contrary to the findings reported in Christoforakos et al. (2021). However, anthropomorphism, perceived warmth, and related constructs were not measured in this study. Therefore, this interpretation should be treated with caution, and future research should explicitly examine how robot embodiment and the perception of social cues interact with politeness and performance in shaping trust.

### 6.5 Limitations

In this work, our primary objective was to implement and evaluate Lakoff’s politeness framework within a controlled experimental setting. Cultural variation may have influenced the interpretation of the rules, which represents an inherent limitation of this study. Moreover, the evaluation was conducted exclusively with Hebrew-speaking engineering participants, potentially constraining the generalizability of our findings.

The participant sample was intentionally restricted to a relatively homogeneous population of young adults with engineering backgrounds within a specific cultural and linguistic context. This design choice reduced variability and enabled a controlled examination of the effects of politeness and erroneous behavior, thereby complementing prior studies conducted with different populations. However, such homogeneity also limits the generalizability of the findings. Because politeness is socially learned and culturally situated, participants’ interpretations of polite behavior, robot errors, and system expectations may reflect context-specific perceptions rather than universal responses in human–robot interaction. Additionally, individuals with engineering backgrounds may emphasize functional performance differently from social aspects of interaction; as this factor was not directly measured, related interpretations should be made cautiously.

Another limitation is the absence of a formal manipulation check assessing whether participants perceived the robot’s behavior as polite or erroneous. Although manipulation checks can provide useful validation and valuable insights, explicitly querying politeness could have revealed the study’s purpose. Given the within-subjects design, such awareness might have introduced demand characteristics and influenced participant responses. Given the within-subjects design, such awareness might have introduced demand characteristics McCambridge et al. (2012) and influenced participant responses. Instead, the study prioritized preserving internal validity. Notably, the observed differences between the polite-correct and non-polite-correct conditions in the mobile robot study suggest that the politeness manipulation was effective when the robot behaved correctly, reducing the likelihood that the results stem from a failed manipulation.

Text-based communication was intentionally employed to maintain strict experimental control over the politeness manipulation and to avoid confounding factors such as prosody, tone, or speech synthesis quality, which could independently affect perceptions of politeness. Presenting the robot’s responses through a graphical interface ensured that differences between conditions were limited to the linguistic politeness cues derived from politeness theory.

Finally, trust was assessed using a two-item measure. As trust is inherently multidimensional, encompassing both cognitive (performance-based) and affective (socially) components, a more comprehensive trust scale could provide deeper insight into how politeness and errors differentially influence these dimensions.

## 7 Conclusions and future work

We conducted a study in which participants, serving in supervisory roles, interacted with two types of robots—a mobile robot and a manipulator—under four behavioral conditions defined by correctness (correct vs. erroneous) and politeness (polite vs. No-rules polite). Our findings emphasize the importance of evaluating HRI performance across different robot types and tasks, as these factors significantly shape user perceptions. More importantly, the results highlight the value of investigating multiple HRI measures in combination, as such integrative approaches provide deeper insights into the user experience and the dynamics of human-robot collaboration. We conducted a repeated-measures experiment with two manipulated factors: robot correctness and politeness. The results indicated that robots exhibiting both correct and polite behavior were consistently preferred across both robot types and tasks, as reflected in all three dependent measures. In contrast, polite behavior did not mitigate negative perceptions when the robot acted erroneously and received less sympathy, suggesting that while politeness is generally valued, it cannot compensate for performance failures. In some cases, politeness in the presence of errors may even irritate users. This finding should be further studied across diverse populations, robot types, and tasks, particularly when comparing scenarios with low versus high error costs.

Our study has two key implications:

1. Correct robot behavior is critical for positive user perception, particularly in functional task scenarios like those used in this study. The findings highlight the contingent nature of human responses to robots, emphasizing the need for further research that replicates and extends these results across a wider variety of robot types and task contexts.

2. Politeness has the potential to enhance user perception, especially when paired with correct behavior. However, it may also backfire when a robot behaves erratically, as elevated expectations created by politeness may not be met, leading to user disappointment. This suggests that robot designers and developers should carefully manage user expectations and avoid overstating a robot’s capabilities.

While employing two different robot types highlights the importance of considering robot type in future research, it is equally crucial to recognize that user perception of robot behavior—especially politeness—is influenced by a range of other factors. Notably, users’ cultural and social backgrounds, as well as personal characteristics like age and education, play a role Lakoff and Ide (2005). It is important to note that the studies reported here involved homogeneous samples of engineering students within a specific cultural context. We expect the findings to be enhanced with non-professional users. Furthermore, while some studies Engelhardt et al. (2017); Cahya and Giuliani (2021); Mahmood et al. (2022) have examined the use of politeness as a mitigating strategy following a robot’s fault, the focus of our study was on robots exhibiting polite (or No-rules polite) behavior consistently throughout the interaction, regardless of performance, and how users perceive them under these conditions.

To enhance the generalizability of politeness research in HRI, future studies should include more diverse participant populations and explicitly account for individual differences, such as user personality traits Besser et al. (2026). Another important consideration is the variety of error types: this study focused on only one kind of error, so future research should investigate different error types Honig and Oron-Gilad (2018) and their potentially distinct impacts on user perception. Furthermore, our study placed humans as supervisors or operators; subsequent work should examine other interaction dynamics, such as scenarios where humans and robots act as equal peers. While this work primarily assessed task performance as a measure of success, future research should incorporate more objective behavioral metrics and qualitative methods, such as interviews, to gain deeper insights into the human-robot interaction experience.

## Data Availability

The raw data supporting the conclusions of this article will be made available by the authors, without undue reservation.
